# Butyrate mediates anti-inflammatory effects of *Faecalibacterium prausnitzii* in intestinal epithelial cells through *Dact3*

**DOI:** 10.1080/19490976.2020.1826748

**Published:** 2020-10-15

**Authors:** Marion Lenoir, Rebeca Martín, Edgar Torres-Maravilla, Sead Chadi, Pamela González-Dávila, Harry Sokol, Philippe Langella, Florian Chain, Luis G. Bermúdez-Humarán

**Affiliations:** a Université Paris-Saclay, INRAE, AgroParisTech, Micalis Institute, Jouy-en-Josas, France; b Sorbonne Universités, INSERM, Centre de Recherche Saint-Antoine, CRSA, AP-HP, Saint Antoine Hospital, Gastroenterology department, F-75012 Paris, France

**Keywords:** Commensal bacteria, *Faecalibacterium prausnitzii*, inflammatory bowel disease, transcriptomic analysis, signaling pathway

## Abstract

The commensal bacterium *Faecalibacterium prausnitzii* plays a key role in inflammatory bowel disease (IBD) pathogenesis and serves as a general health biomarker in humans. However, the host molecular mechanisms that underlie its anti-inflammatory effects remain unknown. In this study we performed a transcriptomic approach on human intestinal epithelial cells (HT-29) stimulated with TNF-α and exposed to *F. prausnitzii* culture supernatant (SN) in order to determine the impact of this commensal bacterium on intestinal epithelial cells. Moreover, modulation of the most upregulated gene after *F. prausnitzii* SN contact was validated both *in vitro* and *in vivo*. Our results showed that *F. prausnitzii* SN upregulates the expression of *Dact3*, a gene linked to the Wnt/JNK pathway. Interestingly, when we silenced *Dact3* expression, the effect of *F. prausnitzii* SN was lost. Butyrate was identified as the *F. prausnitzii* effector responsible for *Dact3* modulation. *Dact3* upregulation was also validated *in vivo* in both healthy and inflamed mice treated with either *F. prausnitzii* SN or the live bacteria, respectively. Finally, we demonstrated by colon transcriptomics that gut microbiota directly influences *Dact3* expression. This study provides new clues about the host molecular mechanisms involved in the anti-inflammatory effects of the beneficial commensal bacterium *F. prausnitzii*.

## Introduction

Inflammatory bowel disease (IBD) is a group of disorders characterized by chronic inflammation in the gastrointestinal tract.^1^^,2^ One potential cause (and/or consequence) of IBD is the disruption of the intestinal ecosystem equilibrium. For example, gut microbiota analysis of Crohn’s disease (a type of IBD) patients revealed markedly lower diversity of Firmicutes (in particular of the *Clostridium leptum* group) compared to healthy individuals.^3^ These assemblages are also relatively poor in *Faecalibacterium prausnitzii*, a major member of the *C. leptum* group and one of the most abundant intestinal bacteria in healthy adults.^4,5^ Because a potential approach to prevent and treat IBD is the oral administration of probiotic and commensal bacteria^6^, *F. prausnitzii* may represent a relevant target for the development of diagnostic, prognostic, or therapeutic tools.

In different pre-clinical models of IBD, *F. prausnitzii* efficiently improves intestinal inflammation^7,8^ and gut barrier function.^9^ Indeed, through secreted metabolites this bacterium is able to block NF-κB activation and IL-8 production, which both contribute to inflammation.^10^ In addition, this species produces high quantities of butyrate,^4^ a short-chain fatty acid (SCFA) which is important in gut physiology.^11,12^ *F. prausnitzii* also produces several bioactive molecules that affect inflammation and gut barrier function such as shikimic and salicylic acids^12^ and a microbial anti-inflammatory molecule (MAM^13^). Despite many advances in the study of *F. prausnitzii*’s anti-inflammatory effects within the host^4^, we still do not understand the exact molecular mechanism of these effects, which could represent targets for new therapies. To identify the host receptor and signaling pathways involved in the beneficial effects of this anti-inflammatory bacterium, we performed DNA chip-based transcriptomic analyses in human intestinal epithelial cells (IECs; *i.e.*, HT-29 cells) that were stimulated with the proinflammatory cytokine TNF-α and exposed to *F. prausnitzii* culture supernatant (SN). These analyses led us to focus on *Dact3*, a member of the Disheveled Binding Antagonist of beta Catenin (DACT) gene family which negatively regulates the Wnt/JNK signaling pathway.^14^ We present here evidence from both *in vitro* and *in vivo* experiments which provide the first clues about the important role of *Dact3* in the host molecular mechanisms involved in the anti-inflammatory effects of the beneficial commensal bacterium *F. prausnitzii*.

## Results

### Transcriptomic analysis reveals *Dact3* as a target of *F. prausnitzii*

**Figure 1.**
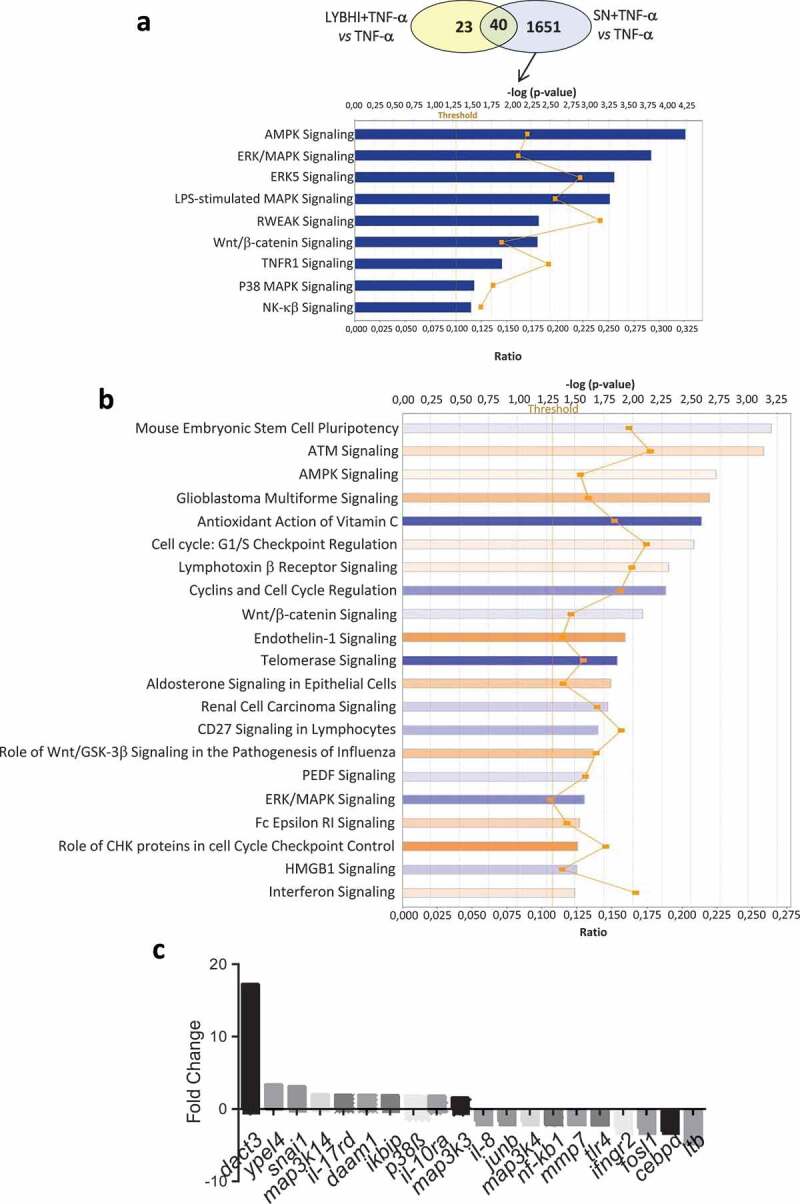
Gene and cellular pathways modulation by *F. prausnitzii* SN. (a) Comparison of the genes modulated by LYBHI+TNF-α *vs* TNF-α and SN+TNF-α *vs* TNF- α. The genes specifically modulated by SN and linked to inflammatory pathways have been represented using IPA canonical pathway display: y-axis displays the -log of the *p-*value which is calculated by Fisher’s exact test right-tailed. The orange points interconnected by a thin line represent the ratio. This ratio is calculated as follows: # of genes in a given pathway that meet the cutoff criteria, divided by the total # of genes that make up that pathway and that are in the reference gene set. (b) IPA canonical pathway display of the genes modulated in the comparison LYBHI+TNF-α *vs* SN+TNF-α: y-axis displays the -log of *p-*value which is calculated by Fisher’s exact test right-tailed. The orange and blue colored bars indicate predicted pathway activation, or predicted inhibition, respectively (*z-*score). Only genes with *z-*score are represented. The orange points interconnected by a thin line represent the ratio. (c) Histogram of the ten most up- and downregulated genes in the comparison LYBHI+TNF-α*vs* SN+TNF-α

**Figure 2.**
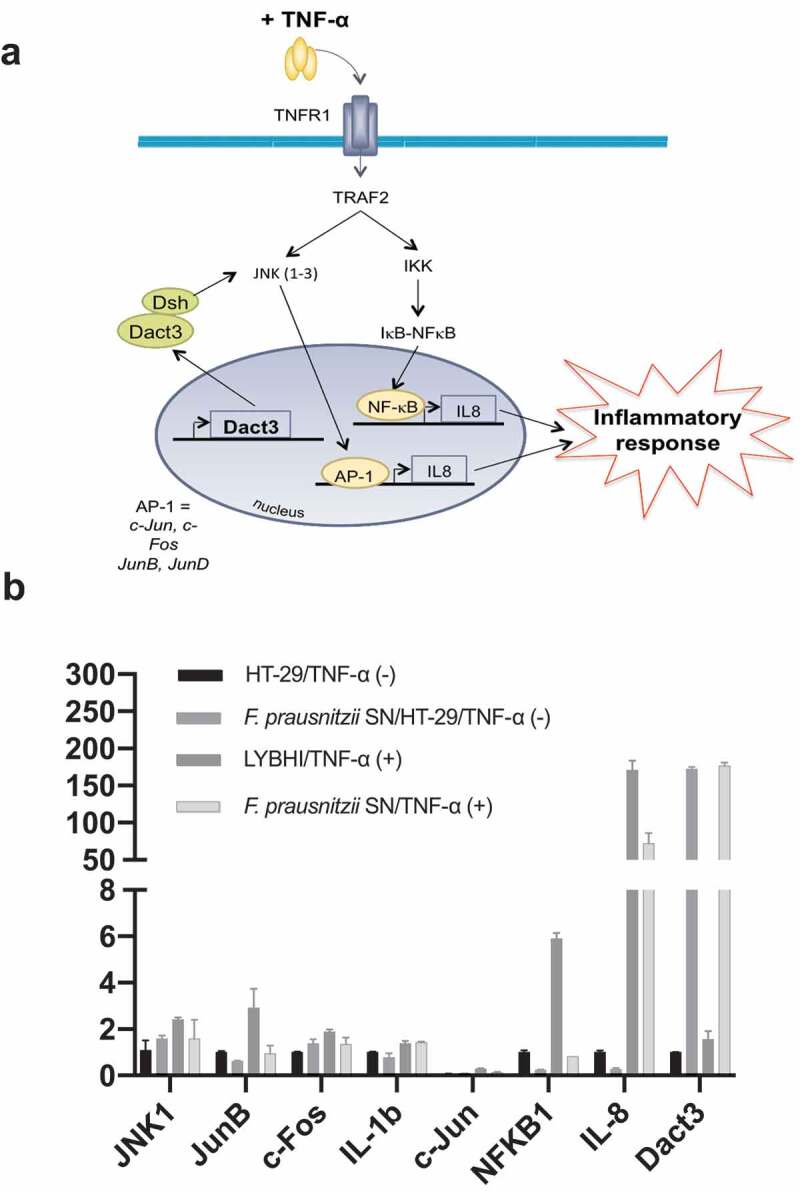
*F. prausnitzii* SN modulates *Dact3* expression *in vitro*. (a) Schematic representation of *Dact3* pathway. (b) RT-qPCR validation of most up- and downregulated genes by *F. prausnitzii* SN in TNF-α-stimulated HT-29 cells. Results are expressed as the fold change (FC) of gene expression relative to the *B2M* housekeeping gene

We used TNF-α to stimulate inflammation in HT-29 cells. A specific trait of this stimulation is upregulation of IL-8^15–19^, which we then measured as a readout for cell inflammatory status. To extend previous research on *F. prausnitzii* SN in TNF-α-stimulated HT-29 cells^12^, we performed a SN dose-effect experiment and determined the stability (i.e., putative degradation) of both IL-8 and TNF-α in the presence of the SN. *F. prausnitzii* SN had a significant dose–response effect (5% to 30%) while the bacterial culture medium LYBHI did not (Figure S1(a)). Of note, *F. prausnitzii* SN did not directly degrade either IL-8 (Figure S1(b)) or TNF-α (Figure S1(c)). To elucidate the host molecular mechanisms underlying the anti-inflammatory effects of *F. prausnitzii* (e.g., specifically the modulatory effects on IL-8 production), we performed a transcriptomic analysis of TNF-α-stimulated HT-29 cells exposed to *F. prausnitzii* SN (microarray hybridization schema is presented in Figure S2). Before evaluating the effects of the SN, we first determined the regulatory changes due to TNF-α stimulation alone. Compared to control HT-29 cells, 227 genes were upregulated and 60 genes were downregulated in TNF-α-stimulated cells (adj. *p < .05*, |fold change (FC)| > 1.5). The genes with the largest changes in expression are listed in Table S1. As expected, the pro-inflammatory cytokine IL-8 was among the most upregulated genes. In addition, upstream regulators such as TNF-α and IFN-γ and signaling pathways such as NF-κB were also activated, confirming the inflamed status of the HT-29 cells. Next, we evaluated the effect of the LYBHI medium used to culture *F. prausnitzii*. Here, only 63 genes were regulated (Figure 1(a)), and LYBHI did not seem to reduce cellular inflammation. Finally, we introduced *F. prausnitzii* SN. There were extensive changes: stimulation and SN treatment specifically modulated 1651 genes (Figure 1(a)). Interestingly, these genes are involved in several inflammatory pathways such as NF-κβ, p38 and other ERK/MAPK pathways. Among these, 25% of the genes that had been activated by TNF-α exposure (in our first comparison) were inactivated by treatment with *F. prausnitzii* SN (70 out of 287). To explore this complex gene regulation network, we submitted the dataset of genes regulated by SN compared to LYBHI to an ingenuity pathway analysis (IPA), which highlighted the SN-affected pathways. This analysis confirmed the ability of *F. prausnitzii* SN to regulate the ERK/MAPK signaling pathway as the most important pathway related to inflammation modulated specifically by *F. prausnitzii* SN (Figure 1(b)). Notably, 7% of all genes regulated by *F. prausnitzii* SN were linked to MAPK pathways, including JNK. The 20 genes with the largest fold-change in regulation are shown in Figure 1(c) and Table S2. IL-8 was found to be downregulated, while the most upregulated gene was *Dact3* (FC = 17.2). *Dact3* belongs to the *Dact* gene family, whose members interact with the Dsh protein to inhibit Dsh-induced activation of the JNK pathway. The *Dact3*/JNK pathway is presented in Figure 2(a). Finally, in order to validate our transcriptomic data, the differential expression of some of the most up- and downregulated genes (see Table S3 for the list of genes analyzed) was validated by RT-qPCR using *B2M* gene as reference for data normalization as previously described.^20^ As shown in Figure 2(b) all the tested genes displayed the same regulation (e.g., up- or down-) as that observed in the transcriptomic analysis. Strikingly, compared to LYBHI, *F. prausnitzii* SN led to significant *Dact3* upregulation in TNF-α-stimulated HT-29 cells, confirming transcriptomic observations.

### Role of Dact3 in TNF-α-stimulated HT-29 cells

**Figure 3.**
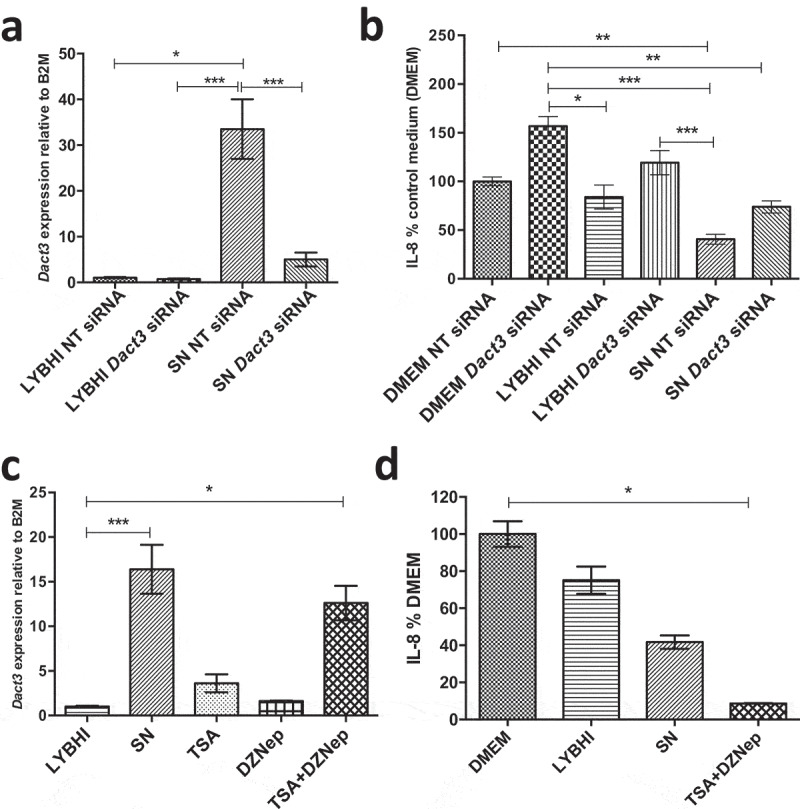
Modulation of *Dact3* expression in TNF-α-stimulated HT-29 cells under different conditions. (a) *Dact3* expression and (b) IL-8 production by HT-29 cells transfected with either *Dact3* or non-targeting (NT) siRNA and treated with both *F. prausnitzii* SN and TNF-α. *Dact3* expression was analyzed by RT-qPCR and IL-8 production by ELISA. Results are represented as the FC of *Dact3* expression relative to LYBHI with NT siRNA and IL-8 have been normalized to DMEM medium (used as a control) under NT siRNA conditions (values for DMEM+TNF-a conditions were set to 100%). (c) *Dact3* expression and (d) IL-8 production by TNF-α-stimulated HT-29 cells co-incubated with 0.5 µM of TSA or 12.5 µM of DZNep, or both. Results are represented as the FC of *Dact3* expression relative to LYBHI and IL-8 values have been normalized to control DMEM. All experiments were performed in triplicate. Non-parametric Kruskal-Wallis and Dunn’s post hoc test *p < .05; **p < .01; ***p < .001

As *Dact3* was the most upregulated gene in the transcriptomics analysis, we thus decided to focus our investigations on this gene. To investigate the effect of *Dact3* on IL-8 production in TNF-α-stimulated HT-29 cells, we overexpressed or knocked down *Dact3* in IECs. First, we tried to overexpress *Dact3* using transient transfection in HT-29 cells; unfortunately, we were unable to efficiently transfect these cells (data not shown). This could be due to the strong upregulation of *Dact3* that led to massive apoptosis of the cells, as was previously reported for colorectal cancer cells.^21^ We then used the siRNA technology to knockdown *Dact3* mRNA in TNF-α-inflamed HT-29 cells. As shown in Figure 3(a), RT-qPCR analysis confirmed that: i) both the non-targeting (NT) siRNA and LYBHI had no effect on *Dact3* expression (FC = 1); ii) *F. prausnitzii* SN strongly upregulated *Dact3* (FC = 30) in presence of NT siRNA; and iii) *Dact3* siRNA significantly reduced the abundance of *Dact3* mRNA induced by *F. prausnitzii* SN. In addition, we confirmed knockdown of *Dact3* at level protein by Western blot analyses as shown in Figure S3. Besides, treatment with *Dact3* siRNA in TNF-α-stimulated HT-29 cells tends to increase IL-8 production (Figure 3(b)) suggesting an important role of *Dact3* activation in intestinal homeostasis. Moreover, the inhibitory effect of *F. prausnitzii* SN on IL-8 production was partially lost in *Dact3*-silencing condition siRNA.

Next, we evaluated the effect of factors modulating *Dact3* expression on IL-8 production. As histone modification has been reported to modulate *Dact3* expression^21^, we used a mix of TSA (a histone deacetylase inhibitor) and DZNep (a histone methylation inhibitor) drugs as previously described.^21^ These drugs led to a strong upregulation of *Dact3* similar to that obtained with *F. prausnitzii* SN (Figure 3(c)). Furthermore, after drug treatment and TNF-α stimulation, *Dact3* upregulation strongly inhibited IL-8 production in HT-29 cells, to an even stronger extent than was observed with *F. prausnitzii* SN (Figure 3(d)).

### *Identification of the* F. prausnitzii*-effectors responsible for Dact3 modulation*

**Figure 4.**
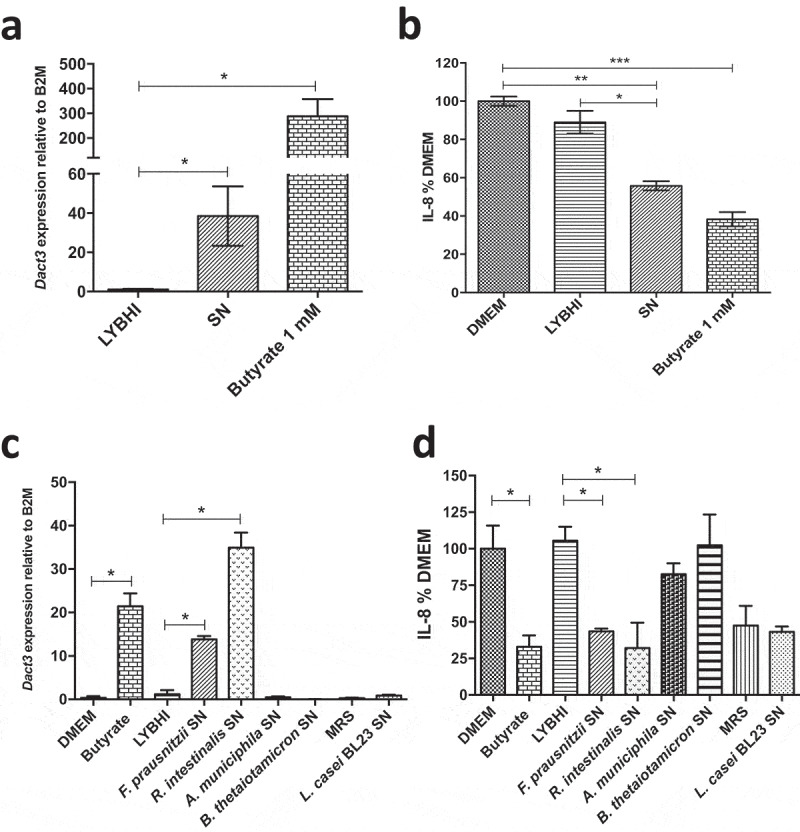
Effect of butyrate on *Dact3* expression. (a) *Dact3* expression and (b) IL-8 production by HT-29 cells co-incubated with 1 mM of butyrate. Results are represented as the FC of *Dact3* expression relative to LYBHI and IL-8 have been normalized to DMEM. All experiments were performed in triplicate. Non-parametric Kruskal-Wallis and Dunn’s post hoc test *p < .05; **p < .01; ***p < .001. (c) *Dact3* expression and (d) IL-8 production in TNF-α-stimulated HT-29 cells and treated with the SN of different bacterial strains producing or not butyrate. LYBHI and MRS were used as negative control of the SN from the different bacterial strains. Results are expressed as the FC of *Dact3* expression relative to LYBHI and IL-8 have been normalized to DMEM. All experiments were performed in triplicate. Non-parametric Kruskal-Wallis and Dunn’s post hoc test *p < .05

To identify the bacterial effector(s) responsible for *Dact3* modulation, as *F. prausnitzii* abundantly produces butyrate, which reduces IL-8 production in TNF-α-stimulated HT-29 cells^12^, we decided to test the effect of this SCFA on *Dact3* modulation. As shown in Figure 4(a), treatment with 1 mM of butyrate (the concentration present in the *F. prausnitzii* SN^22^) led to strong upregulation of *Dact3* and a decrease in IL-8 production (Figure 4(b)). Altogether, these results reveal that butyrate is one of the *F. prausnitzii* produced-metabolites responsible for *Dact3* modulation.

In order to validate this hypothesis, we tested other gut bacterial strains (*e.g. Roseburia intestinalis, Akkermansia muciniphila* and *Bacteroides thetaiotaomicron*), known to produce or not butyrate (Table S4), for their capacities to upregulate *Dact3* (Figure 4(c)) and downregulate IL-8 (Figure 4(d)). We also tested a probiotic lactic acid bacterium (LAB) with anti-inflammatory capacities, *L. casei* BL23, that does not produce butyrate. Strikingly, the SN from the only other butyrate-producing bacterium (*R. intestinalis*) highly upregulated *Dact3* expression and thus downregulated IL-8 production. In contrast, the three other bacterial strains that do not produce butyrate (*A. muciniphila, B. thetaiotaomicron* and *L. casei* BL23) did not have any effect on neither *Dact3* nor IL-8. Despite *L. casei* BL23 seems to reduce IL-8 levels, this effect is more due to its culture medium (*e.g*. MRS) than the bacterium itself since the same inhibition in IL-8 levels was observed with the medium alone. This phenomenon could be explained by the presence of serpin in MRS, a protease inhibitor well known to display immunomodulatory effects.^23^

### In vivo *validation of* Dact3 *modulation*

**Figure 5.**
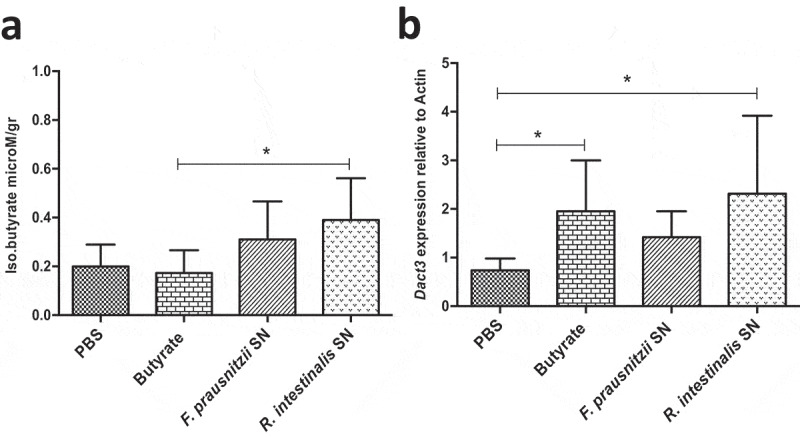
Modulation of *Dact3* expression *in vivo* by *F. prausnitzii* SN. Animals were intragastrically administered with either butyrate (1 mM) *F. prausnitzii* SN or *R. intestinalis* SN and sacrificed 9 after. (a) Quantification of butyrate in samples from proximal colon of treated mice. (b) FC of *Dact3* expression in colonic samples relative to actin housekeeping gene. Non-parametric Kruskal-Wallis and Dunn’s post hoc test *p < .05

*Dact3* modulation was first investigated in healthy mice orally administered with *F. prausnitzii* SN and sacrificed at different time points (*e.g*. 0, 3, 6, and 9 hours after administration) to evaluate its effects in normal physiological conditions (Figure S4(a)). *F. prausnitzii* SN led to a significant increase in *Dact3* mRNA in colonic samples 9 h after its administration (Figure S4(b)). Other groups of mice were orally administered with either *F. prausnitzii* SN, *R. intestinalis* SN or butyrate (1 mM) and euthanized 9 h later. As shown in Figure 5(a), butyrate was detected in colonic samples of mice treated with either *F. prausnitzii* SN or *R. intestinalis* SN but not with butyrate itself, suggesting that soluble butyrate is rapidly absorbed in the colon. Strikingly, all treatments, including butyrate, results in *Dact3* upregulation in samples from proximal colon (Figure 5(b)).

**Figure 6.**
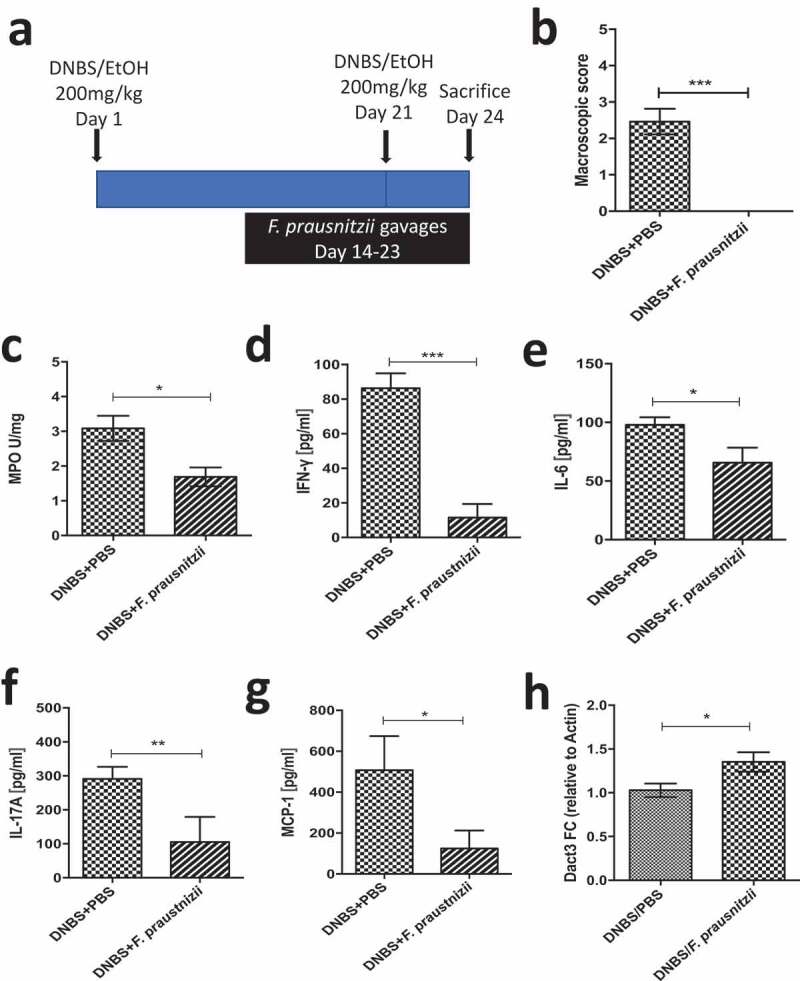
Modulation of *Dact3* expression *in vivo* by *F. prausnitzii*. (a) Experimental protocol used for the analysis of the *in vivo* effects of *F. prausnitzii* in a mouse model of chronic inflammation (as previously described^8^): (b) Macroscopic scores; (c) MPO activity; (d–g) colonic pro-inflammatory cytokine and chemokine concentrations and (h) FC of *Dact3* expression in colonic samples relative to actin housekeeping gene. Non-parametric Mann-Whitney U test *p < .05

We then investigated *in vivo Dact3* modulation in an inflammatory context, using a murine model in which chronic moderate inflammation was induced by intrarectal injection of dinitrobenzene sulfonic acid (DNBS). Animals were given daily oral gavages of live *F. prausnitzii* (Figure 6(a)), as previously described.^8^ As expected, *F. prausnitzii* administration led to significant reductions in markers of colitis with improvements in weight loss and a decrease in macroscopic scores (Figure 6(b)), MPO activity (Figure 6(c)) and a reduction of the pro-inflammatory cytokines IFN-γ (Figure 6(d)), IL-6 (Figure 6(e)), IL-17A (Figure 6(f)) and the chemokine MCP-1 (Figure 6(g)). Moreover, *Dact3* expression was upregulated in the colon of *F. prausnitzii*-treated mice compared to the PBS-treated mice (Figure 6(h)), confirming that *Dact3* is induced by *F. prausnitzii* in an inflammatory context too.

**Figure 7.**
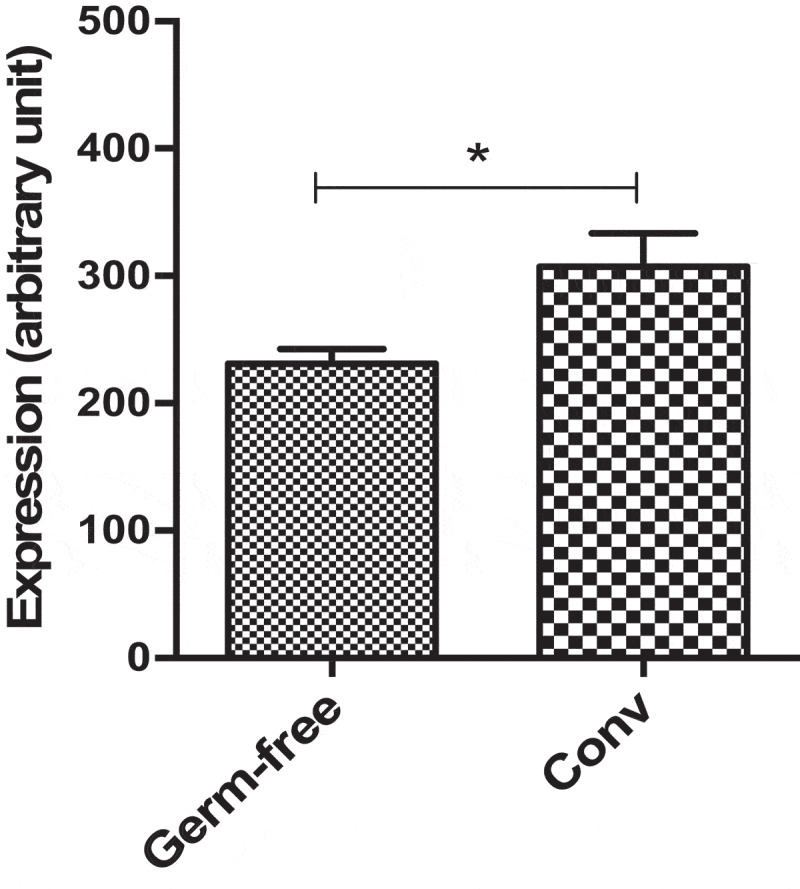
*Dact3* modulation by endogenous microbiota. Total RNA was extracted from colon tissues of both germ-free and conventional (Conv) mice and analyzed by microarrays to determine modulation of *Dact3.*

Finally, to assess the effect of the gut microbiota on *Dact3* modulation in *in vivo* physiological conditions, we investigated a dataset that we previously published comparing the colonic transcriptome of germ-free and conventionalized (Conv) mice.^24^ We found that *Dact3* expression was significantly reduced in germ-free compared to Conv mice (Figure 7). These observations confirm that gut microbiota has a major impact on *Dact3* expression.

## Discussion

*F. prausnitzii* is a commensal bacterium well known for its immunomodulatory properties and more specifically for its anti-inflammatory effects both *in vitro*^7,12,13^ and *in vivo*.^4,7,9,12,13,25^ Here, we demonstrated that *F. prausnitzii* SN is able to block IL-8 production in TNF-α-activated HT-29, but this effect is not due to  the proteolytic degradation of either TNF-α or IL-8. Thus, and because *F. prausnitzii* is extremely oxygen-sensitive (EOS) and cannot be easily cultured with human cells^26^, we decided to further study the immunomodulatory effects of its SN. *F. prausnitzii* SN modulated the expression of a massive number of genes, in fact, more than the TNF-α treatment itself (Table S1). Among the inflammation-related pathways regulated by *F. prausnitzii* SN, we found enrichment in genes related to MAPKs. In particular, *Dact3*, a gene implicated in Wnt/JNK regulation, was among the top-upregulated genes by *F. prausnitzii* SN in non-stimulated as well as in TNF-α-stimulated cells. The *Dact* gene family was initially reported in studies of embryonic development.^14,27^ Although *Dact3* is involved in postnatal development (adult *Dact3^−/-^* mice show a mild reduction in body weight), the fact that *Dact3^−/-^* mice are viable means that this gene is not essential for mouse embryogenesis, postnatal survival, and reproduction.^28^ *Dact3* has a postnatal role as a regulator in the Wnt/β-catenin signaling pathway^21^ and has been associated with several types of cancer: colorectal^21^, breast^29^, ovarian^30^, lung^31^, papillary thyroid^32^, and renal fibrosis.^28^ In colorectal cancer cell lines, *Dact3* transcription is epigenetically downregulated by a bivalent histone modification.^21^ In particular, drugs that target both histone methylation (DZNep) and deacetylation (TSA) strongly induce *Dact3* expression^21^, a result that we confirmed here. Moreover, we also observed that these drugs reduced IL-8 production by TNF-α-stimulated HT-29 cells in a way that was similar to the effect of *F. prausnitzii* SN. These results show that *Dact3* has an important function in the IL-8 pathway and that IL-8 inhibition by *F. prausnitzii* SN is, at least partly, mediated via *Dact3* upregulation. *Dact3* could thus be an epigenetic regulator of inflammation and play a key role in intestinal homeostasis. To further decipher this, we evaluated the effect of *Dact3* silencing in HT-29 using siRNA. Silencing of *Dact3* in IECs prevented *F. prausnitzii* SN from blocking IL-8 production.

*Dact3* histone modulation could provide clues about the bacterial effectors responsible for this regulation. In this study, only butyrate was able to both upregulate *Dact3* expression and to block IL-8 production by TNF-α-stimulated HT-29 cells. Indeed, butyrate-induced reduction in IL-8 production was similar to that observed with the *F. prausnitzii* SN, but *Dact3* upregulation was higher in the presence of butyrate than of SN. This is probably because *Dact3* is also modulated by histone deacetylases^21^ which are known to be inhibited by butyrate.^11^ In this context, our results confirm those obtained by Fung et al.^33^, who found that *Dact3* appeared to be differentially modulated in HT-29 cells treated with butyrate. However, these authors did not further explore *Dact3* in their study.

Altogether, our results provide evidence that *F. prausnitzii* SN regulates *Dact3*/IL-8 production and suggest that butyrate produced by *F. prausnitzii* is the main actor in this regulation. Butyrate is well known to display immunoregulatory effects on IECs; however, these effects are dose and time-dependent in addition to be dependent of the IEC used *in vitro* (e.g., Caco-2 or HT-29 cells). Indeed, in an interesting study the authors found that butyrate decreases IL-8 secretion in IL-1β-stimulated Caco-2 cells, but increases IL-8 production in HT-29 cells, all of intestinal origin.^34^ In another study, it was shown that long-term incubations (~24 h) result in a proinflammatory profile of butyrate in HT-29 cells in an NF-κβ-SEAP reporter system stimulated by TNF-α^35^ while butyrate has been shown to inhibit TNF-α-induced nuclear translocation of NF-κβ (a pro-inflammatory transcription factor) after 30 minutes of TNF- α stimulation.^36^ Thus, as butyrate displays important pleiotropic effects in the intestinal cell life cycle and numerous beneficial effects for human health^11^, it is plausible that butyrate production is a means by which *F. prausnitzii* affects its host physiological functions and homeostasis to maintain health. However, further studies are necessary to confirm this hypothesis. In particular, genetic manipulation of *F. prausnitzii* to inactivate the gene encoding the butyril-CoA synthase involved in butyrate metabolism would be helpful to answer to this question. Indeed, O’Cuiv et al.^37^ have isolated *F. prausnitzii* transconjugants using metaparental mating and this strategy opens promising perspectives to manipulate *F. prausnitzii.*

Finally, *in vitro* observations were validated *in vivo* in two different models: first, in healthy mice that were orally administered *F. prausnitzii* SN one time (Figure S4) and second, in inflamed mice that were given live *F. prausnitzii* orally for 10 days (Figure 7). In both *in vivo* models, either *F. prausnitzii* or *F. prausnitzii* SN positively regulated *Dact3*, confirming the key effect of *F. prausnitzii* on this gene.

In conclusion, we propose in this study a new role for *Dact3* as a master regulator of intestinal homeostasis, particularly in inflamed cells. Although it is expressed in IECs at a low level, *Dact3* seems to be essential for intestinal homeostasis, as its downregulation or loss leads to a global increase in inflammation. We hypothesize that *Dact3* upregulation inhibits the AP-1 transcription factor, which in turn leads to a global downregulation of genes encoding for pro-inflammatory cytokines such as IL-2, IL-6, and IL-8.^38^ Additionally, the ability of *F. prausnitzii* SN to modulate other genes involved in cancer pathways (as revealed in the transcriptomic analysis) represents a novel potential beneficial effect of this commensal anti-inflammatory bacterium, which is currently being investigated by our laboratory.

Our study provides the first clues on one of the host molecular targets involved in the anti-inflammatory effects of *F. prausnitzii* in IECs. Moreover, these results point out *Dact3* as a potential master regulator of inflammation in IECs. As there is increasing interest in exploring new alternatives for IBD treatment, this research suggests at least three potential opportunities: i) use of *F. prausnitzii* itself, ii) use of drugs to modulate *Dact3* expression (such as histone deacetylases) and iii) heterologous delivery of *Dact3* (either as a cDNA or a protein) using food-grade live vectors.^39,40^ For these, further studies on *Dact3*-knockout mice will be necessary to understand the physiological functions of *Dact3*.

## Materiel and methods

### Bacterial strains

*Faecalibacterium prausnitzii* strain A2-165 (DSM N°17677, DSMZ collection, Braunschweig, Germany) was grown in LYBHI medium (BHI, Difco, Detroit, USA) supplemented with 0.5% yeast extract, 1 mg/ml cellobiose (Sigma-Aldrich Chemie GmbH, Buchs, Switzerland), 1 mg/ml maltose and 0.5 mg/ml cysteine (Sigma-Aldrich), at 37°C in an anaerobic chamber. *F. prausnitzii* supernatant (SN) was recovered by centrifugation and filtered through 0.45-μm-pore-size filters (VWR, Haasrode, Belgium) and stored at −80°C.

### Cell lines and co-incubations

The human colon carcinoma cell line HT-29 (ATCC HTB-38) was grown in Dulbecco’s Modified Eagle’s minimal essential medium with 4.5 g/L glucose (DMEM) (Sigma-Aldrich), supplemented with 10% (w/v) heat-inactivated fetal calf serum (FCS) (GibcoBRL, Eragny, France), 4 mM L-glutamine, and penicillin G/streptomycin (5000 IU/mL, 5000 µg/mL) (Sigma-Aldrich). Cultures were incubated in 25-cm^2^ tissue culture flasks (Nunc, Roskilde, Denmark) at 37ºC in a 10% (v/v) CO_2_ atmosphere until confluence.

For co-culture experiments, HT-29 cells were seeded in 24-well culture plates (Nunc) in DMEM supplemented with 10% heat-inactivated FCS-1% glutamine at 37°C in a 10% CO_2_-air atmosphere. Culture medium was changed every day. Experiments began on day 7 after seeding, when cells were at confluence (approx. 1.83 × 10^6^ cells/well). On day 6, 24 h before co-culture with *F. prausnitzii* SN, the culture medium was changed to one with 5% heat-inactivated FCS and 1% glutamine. The day of the co-culture, either SN or LYBHI medium was added at a concentration of 10% (v/v) in a total volume of 500 µl. Cells were stimulated simultaneously with recombinant human TNF-α (5 ng/ml; Peprotech, NJ, USA) for 6 h at 37°C in 10% CO_2_. All samples were analyzed in triplicate.

### ELISA

Supernatants of mock non-stimulated and TNF-α-stimulated HT-29 cells used for transcriptomic analysis were first validated for IL-8 modulation by ELISA. Supernatants for ELISA and cells for RNA extractions and transcriptomic analysis were collected at the same time and from the same culture plates. IL-8 concentrations were determined by ELISA (Biolegend, San Diego, CA), according to the manufacturer’s instructions. Results were reported as the mean values of duplicate ELISA wells.

### RNA isolation

After co-incubation with either LYBHI or *F. prausnitzii* SN, HT-29 cells were collected and RNA-purified for DNA transcriptomic hybridization. Total RNA was extracted from cells using the RNeasy Mini Kit (Qiagen, USA) and purified by on-column digestion of DNA with DNase I as recommended by the manufacturer to eliminate residual genomic DNA. RNA concentration was determined by Nanodrop quantification (Thermo Fisher Scientific Inc., France). RNA quality was checked on an Agilent 2100 Expert Bioanalyzer (Agilent Technologies, France). Only RNAs with a RIN > 8 were used for transcriptomic and qRT-PCR experiments.

### Microarray hybridization

A reference design with complete dye-swap including two biological replicates was used to compare HT-29 cells among different treatments, for a total of 6 Agilent 4x44K Whole Human Genome Microarrays (Agilent Technologies, France). Scheme design is available in Figure S2. For labeling, 100 ng of total RNA was reverse-transcribed and stained with Cy3 or Cy5 using the two-color Low Input Quick Amp Gene Expression Labeling Kit (Agilent Technologies, France) according to the manufacturer’s instructions. The CyDye-labeled cRNAs were then purified using the RNeasy Mini Kit (Qiagen, France). cRNA quantity was determined by Nanodrop and quality was checked on the Agilent 2100 Expert Bioanalyzer. Yield and specific activity were determined per manufacturer’s instructions. We mixed 825 ng of Cy3-labeled cRNA from one treatment with the same amount of Cy5-labeled cRNA from another treatment. cRNAs were hybridized to the Agilent 4x44K Whole Human Genome Microarray (Agilent Technologies, France) at 65°C for 17 h in a rotating incubator. After hybridization, slides were washed and then scanned using an Agilent G2565CA scanner (Agilent Technologies, France). Raw data were extracted using Feature Extraction software version 10.5.1.1 (Agilent Technologies, France).

### Microarray analysis

R 3.0.2 software and the LIMMA package^17^ were used to analyze microarray data (within-array normalization by loess method followed by between-array normalization by quantile method) and to generate lists of differentially expressed genes. Microarray data were deposited in the NCBI-GEO database (accession number GSE72048). To create gene lists, we filtered by expression levels (|(FC)| > 1.5 as a cutoff) as well as by adjusted *p-*values, using a 0.05 threshold with the Benjamini and Hochberg false discovery rate^18^ as multiple testing correction. Selected gene lists (log ratio and *p-*value data) were loaded into Ingenuity Pathway Analysis (IPA) and Multiarray Experiment Viewer^41^ to analyze pathways and generate data displays.

### Quantitative real-time RT-PCR (qRT-PCR)

Three μg of DNase I-treated total RNAs were reverse-transcribed using Oligo(dT) primers and 1 µl of SuperScript II reverse transcriptase (Invitrogen, France). The resulting cDNAs were quantified by Nanodrop (Thermo Fisher Scientific Inc., France) and diluted to a working concentration of 100 ng/μl. Reactions were performed in a final volume of 25 μl with 500 ng cDNA, 10 pM primers, and SYBR Green PCR Master Mix (Applied Biosystems, USA), using a Mastercycler Realplex (Eppendorf, France). Primers are listed in Table S4. The genes B2M (for human analysis), HMBS or actin (for mice analysis) were used as internal references^42^ and the 2^−ΔΔCT^ method^43^ was used to calculate the FC in gene expression.

### Dact3 siRNA in HT-29 cells

HT-29 cells were cultured as described above. siGENOME® Human Dact3 siRNA-SMARTpool®, Dact3 siRNA D-015690-01, D-015690-02, D-015690-03, D-015690-17, and control siRNA (Non-Targeting siRNA D001136-01-05 and Cyclophilin B D-001210-02-05) siRNAs were transfected into HT-29 cells using Dharmafect 1 Transfection Reagent (Dharmacon, USA) following manufacturer’s instructions with some modifications. A total of 1 × 10^5^ cells were plated in 12-well plates and transfected using 30 nmol siRNA and 2 µL of Dharmafect 1 Transfection Reagent per well in DMEM containing 5% FCS and 1% L-Glutamine. After 24 h, the medium was changed. After another 24 h, control medium, *F. prausnitzii* SN, or LYBHI medium was added at a concentration of 10% (v/v) in a total volume of 1 ml. Cells were simultaneously stimulated with recombinant human TNF-α (5 ng/ml; Peprotech, NJ, USA) at 37°C in 10% CO_2_. After 6 h of co-culture, supernatants were tested for IL-8 production by ELISA and RNA was isolated from cells for *Dact3* expression analysis, as described above. All samples were generated and analyzed in triplicate.

### Dact3 overexpression in HT-29 cells

HT-29 cells were cultured as above. The S-adenosylhomocystein hydrolase inhibitor 3-Deazaneplanocin A (DZNep) and Trichostatin A (Sigma-Aldrich Chemie GmbH, Buchs, Switzerland) were used as described in^21^ with some modifications. Cells were plated at 0.5 × 10^5^ cells/well; 24 h later, 12.5 M DZNep was added, and 48 hours after that, 0.5 µM TSA was added for the final 24 h of culture. After treatment, cells were stimulated with TNF-α (5 ng/ml; Peprotech, NJ, USA) and *F. prausnitzii* SN or LYBHI (added at 10% v/v). After 6 h of co-incubation, supernatants were tested by ELISA for IL-8 production and RNA was isolated from cells for *Dact3* expression analysis. All samples were performed in triplicate.

### Dact3-microbiota modulation in vivo

Microarray data comparing germ-free and conventionalized mice were described and published previously.^24^ GEO accession number: GSE63299 Conventionalization of germ-free C3H/HeN mice was performed with fresh stools from C3H/HeN donor mice.

### Dact3-F. prausnitzii SN modulation in vivo

C57BL/6 mice (males, 6–8 weeks of age; Janvier, Le Genest Saint Isle, France) were maintained at the animal care facilities of the National Institute of Agricultural Research (IERP, INRA, Jouy-en-Josas, France) under specific pathogen-free conditions. Mice were housed under standard conditions for a minimum of 1 week before experimentation. All experiments were performed in accordance with European Community rules and approved by the animal care committee COMETHEA (Comité d’Ethique en Expérimentation Animale du Center INRA de Jouy-en-Josas et AgroParisTech, Jouy-en-Josas, France). All assays were carried out under agreement N°3445-2016010615159974.

The protocol for *Dact3* modulation and quantification *in vivo* is illustrated in Figure S4(a). Three groups of mice (*n = *8) were intragastrically administered 200 µl of *F. prausnitzii* SN and sacrificed 3 h (T3), 6 h (T6) or 9 h (T9) after gavage. Control (T0) mice were not treated. Colitis was induced as described in^4^, by intrarectal injection of 200 mg/kg of DNBS solution (Sigma-Aldrich) in 30% ethanol. Fourteen days following the first injection, a 200-µl solution of either 1 × 10^9^ CFU of *F. prausnitzii* or PBS was administered intragastrically for 10 days. Colitis was reactivated 21 days after the first DNBS injection with a second injection of 200mg/kg of DNBS solution. Mice were sacrificed 3 days after the second DNBS injection (Figure S4(a)) by cervical dislocation and different parameters of inflammation were recorded as previously described.^8^

Colon samples (1 cm from distal colon) were frozen in 500 µl of RNAlater solution (Ambion, France) in liquid nitrogen and stored at −80°C until use. Total RNA was extracted from individual samples with the RNeasy mini kit (Qiagen) according to the manufacturer’s instructions, using homogenization with Tissue Lyser (Qiagen) and purification by on-column digestion of DNA with DNase I. Total RNA was determined by Nanodrop quantification. *Dact3* expression was analyzed by qRT-PCR.

### Statistical analysis

Statistical analysis, with the exception of transcriptomic analysis, was completed using GraphPad (GraphPad Software, La Jolla, CA, USA). A *p-*value of less than 0.05 was considered significant. Significant differences in the relative expression values of the target genes were tested with REST software using pairwise-fixed reallocation randomization.^44^

## Supplementary Material

Supplemental Material
