# Supplementary material for: Butyrate mediates anti-inflammatory effects of Faecalibacterium prausnitzii in intestinal epithelial cells through Dact3
Source: Gut Microbes. 2020 Oct 15;12(1):1826748. doi: 10.1080/19490976.2020.1826748 (PMC7567499; doi:10.1080/19490976.2020.1826748)
Supplement: Supplemental Material [file KGMI_A_1826748_SM0657.zip › Supplementary information/Supplementary figure legends.docx]

**Supplementary figure legends**

**Fig. S1** Immuno-modulatory abilities of *F. prausnitzii* SN *in vitro.* ***(A)*** IL-8 production by TNF-α-stimulated HT-29 cells co-incubated with either PBS, LYBHI bacterial culture medium, or different concentrations of *F. prausnitzii* SN (5% to 30%). Results are expressed as IL-8/protein (pg/mg). All experiments were performed in triplicate. Media of all the % of LYBHI tested (5%, 10%, 15%, 20%, 30%). ***(B)*** Stability of IL-8 in PBS, SN or LYBHI after a 6 hours incubation. ***(C)*** Stability of TNF-a in PBS, SN or LYBHI after a 6 hours incubation. Non-parametric Kruskal-Wallis test **p<* 0.05.

**Fig. S2** Hybridization scheme for the transcriptomic analysis of TNF-α-stimulated HT-29 cells exposed to *F. prausnitzii* SN.

**Fig. S3** Western blot detection of *Dact3* in TNF-α-stimulated HT-29 cells transfected with *Dact3* siRNA and treated with *F. prausnitzii* SN. HT-29 cells were cultured as described in the manuscript (Material and Methods section). siGENOME® Human *Dact3* siRNA-SMARTpool®, Dact3 siRNA D-015690-01, D-015690-02, D-015690-03, D-015690-17, was transfected into HT-29 cells using Lipofectamine™ 3000 Transfection Reagent (Invitrogen) following manufacturer's instructions with some modifications: a total of 1X10^5^ cells were plated in 12-well plates and transfected using 30 nmol siRNA and 2 µL of Lipofectamine™ 3000 per well in DMEM containing 5% FCS and 1% L-Glutamine. After 24 h the medium was changed and 24 h later we added either the control medium, *F. prausnitzii* SN, or LYBHI medium at a concentration of 10% (v/v) in a total volume of 1 ml. Cells were simultaneously stimulated with recombinant human TNF-α (5 ng/ml; Peprotech, NJ, USA) at 37°C in 10% CO_2_. After 6 h of co-culture, HT-29 cells were washed twice with PBS and re-suspended in 250 μl of Passive Lysis Buffer 1X (PLB-Promega) plus protease inhibitors (Roche) and homogenized at 400 rpm during 15 min. Equal amounts of proteins were diluted 1:4 in Laemmli sample buffer containing 355 mM β-mercaptoethanol and denaturized 5 min at 95°C and centrifuged at 6000 *×* g, 15 seconds. Equal amounts of proteins were loaded and separated on a Mini-PROTEAN TGX stain free 4-20% SDS gel at 100V and further transferred to a PVDF membrane (biorad) using a Trans-Blot Turbo transfer system (Biorad). Membrane was probed with primary antibody anti-human Dapper3 (Santa Cruz Biotechnology, USA) at 1:200 dilution and secondary anti-mouse IgG HRP-conjugated antibody (Abliance) and 1:1000 dilution. Bound secondary antibody was visualized by the SuperSignal West Pico PLUS Chemiluminescent Substrate (Thermo Fisher Scientific Inc., France) and Chemidoc imaging system (Biorad).

**Fig. S4** Modulation of *Dact3* expression *in vivo* by *F. prausnitzii* SN. ***(A)*** Experimental protocol used for *in vivo* analysis in healthy mice. Animals were intragastrically administered *F. prausnitzii* SN and sacrificed at different time points (3 h, 6 h, or 9 h). ***(B)*** FC of *Dact3* expression in colonic samples relative to the HMBS housekeeping gene.
