# Supplementary material for: Butyrate mediates anti-inflammatory effects of Faecalibacterium prausnitzii in intestinal epithelial cells through Dact3
Source: Gut Microbes. 2020 Oct 15;12(1):1826748. doi: 10.1080/19490976.2020.1826748 (PMC7567499; doi:10.1080/19490976.2020.1826748)
Supplement: Supplemental Material [file KGMI_A_1826748_SM0657.zip › Supplementary information/Tables Suppl MS Dact3 Gut Microbes.pptx]

## Slide 1
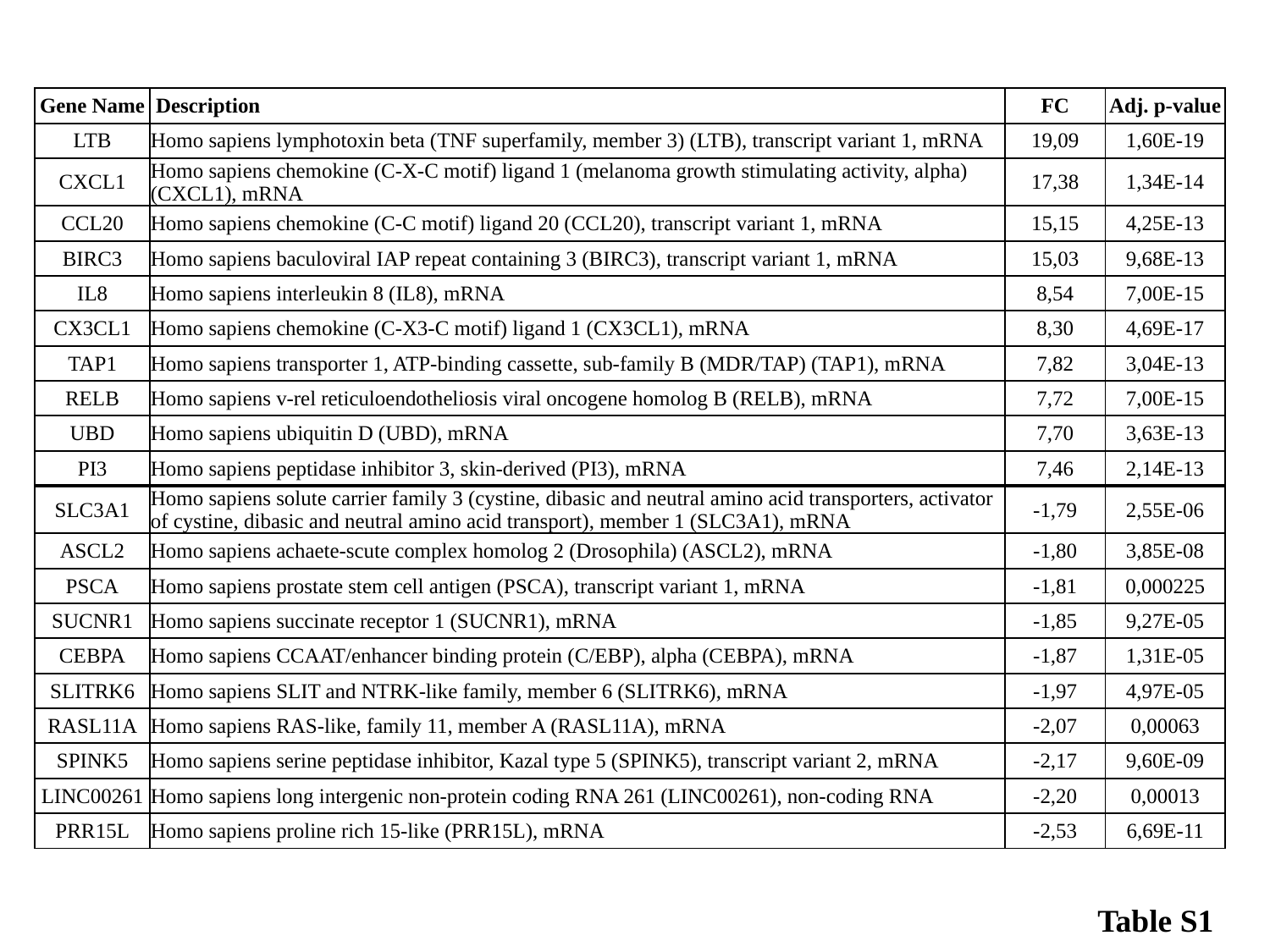

| Gene Name | Description | FC | Adj. p-value |
| --- | --- | --- | --- |
| LTB | Homo sapiens lymphotoxin beta (TNF superfamily, member 3) (LTB), transcript variant 1, mRNA | 19,09 | 1,60E-19 |
| CXCL1 | Homo sapiens chemokine (C-X-C motif) ligand 1 (melanoma growth stimulating activity, alpha) (CXCL1), mRNA | 17,38 | 1,34E-14 |
| CCL20 | Homo sapiens chemokine (C-C motif) ligand 20 (CCL20), transcript variant 1, mRNA | 15,15 | 4,25E-13 |
| BIRC3 | Homo sapiens baculoviral IAP repeat containing 3 (BIRC3), transcript variant 1, mRNA | 15,03 | 9,68E-13 |
| IL8 | Homo sapiens interleukin 8 (IL8), mRNA | 8,54 | 7,00E-15 |
| CX3CL1 | Homo sapiens chemokine (C-X3-C motif) ligand 1 (CX3CL1), mRNA | 8,30 | 4,69E-17 |
| TAP1 | Homo sapiens transporter 1, ATP-binding cassette, sub-family B (MDR/TAP) (TAP1), mRNA | 7,82 | 3,04E-13 |
| RELB | Homo sapiens v-rel reticuloendotheliosis viral oncogene homolog B (RELB), mRNA | 7,72 | 7,00E-15 |
| UBD | Homo sapiens ubiquitin D (UBD), mRNA | 7,70 | 3,63E-13 |
| PI3 | Homo sapiens peptidase inhibitor 3, skin-derived (PI3), mRNA | 7,46 | 2,14E-13 |
| SLC3A1 | Homo sapiens solute carrier family 3 (cystine, dibasic and neutral amino acid transporters, activator of cystine, dibasic and neutral amino acid transport), member 1 (SLC3A1), mRNA | -1,79 | 2,55E-06 |
| ASCL2 | Homo sapiens achaete-scute complex homolog 2 (Drosophila) (ASCL2), mRNA | -1,80 | 3,85E-08 |
| PSCA | Homo sapiens prostate stem cell antigen (PSCA), transcript variant 1, mRNA | -1,81 | 0,000225 |
| SUCNR1 | Homo sapiens succinate receptor 1 (SUCNR1), mRNA | -1,85 | 9,27E-05 |
| CEBPA | Homo sapiens CCAAT/enhancer binding protein (C/EBP), alpha (CEBPA), mRNA | -1,87 | 1,31E-05 |
| SLITRK6 | Homo sapiens SLIT and NTRK-like family, member 6 (SLITRK6), mRNA | -1,97 | 4,97E-05 |
| RASL11A | Homo sapiens RAS-like, family 11, member A (RASL11A), mRNA | -2,07 | 0,00063 |
| SPINK5 | Homo sapiens serine peptidase inhibitor, Kazal type 5 (SPINK5), transcript variant 2, mRNA | -2,17 | 9,60E-09 |
| LINC00261 | Homo sapiens long intergenic non-protein coding RNA 261 (LINC00261), non-coding RNA | -2,20 | 0,00013 |
| PRR15L | Homo sapiens proline rich 15-like (PRR15L), mRNA | -2,53 | 6,69E-11 |
Table S1

## Slide 2
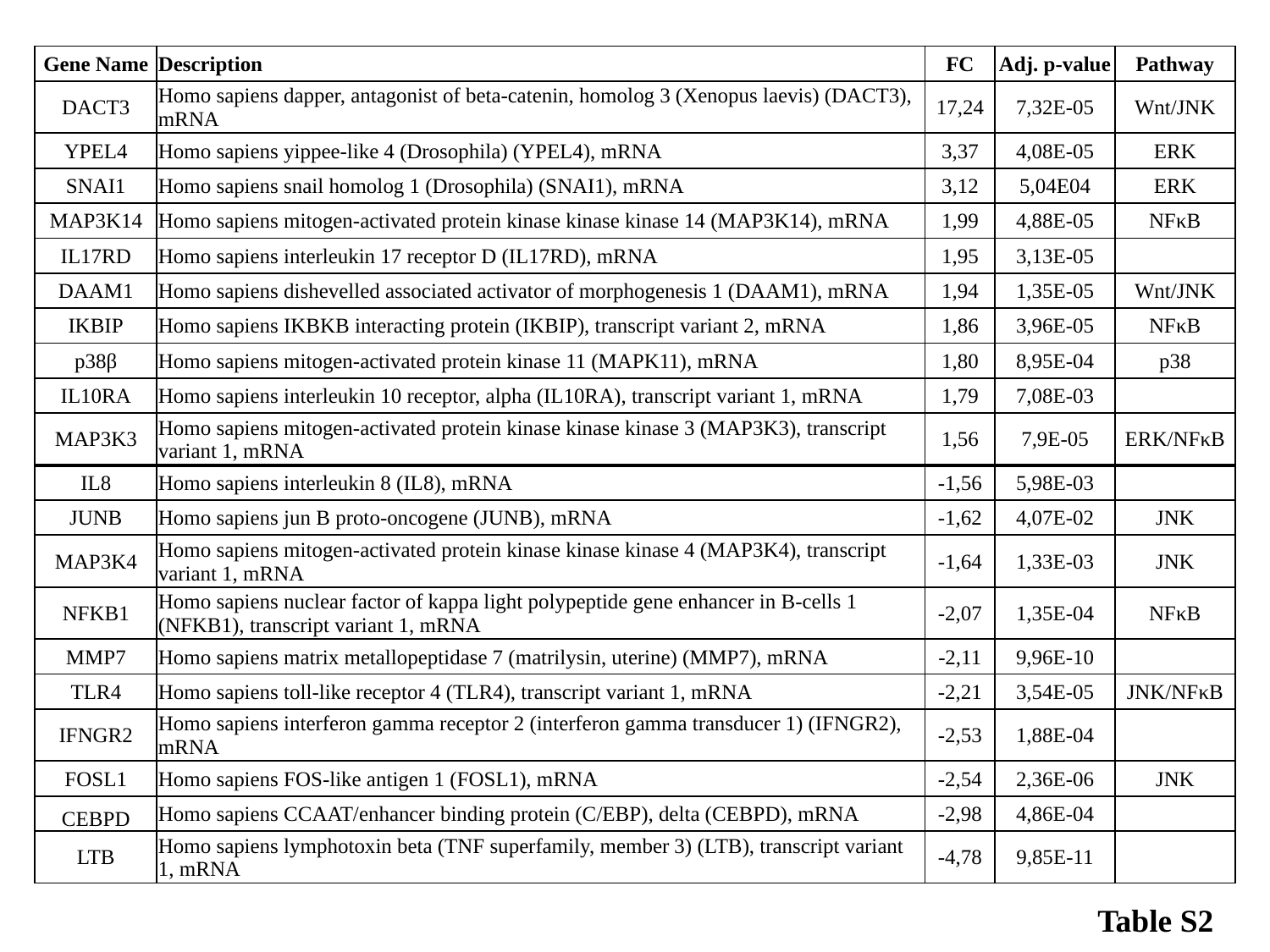

| Gene Name | Description | FC | Adj. p-value | Pathway |
| --- | --- | --- | --- | --- |
| DACT3 | Homo sapiens dapper, antagonist of beta-catenin, homolog 3 (Xenopus laevis) (DACT3), mRNA | 17,24 | 7,32E-05 | Wnt/JNK |
| YPEL4 | Homo sapiens yippee-like 4 (Drosophila) (YPEL4), mRNA | 3,37 | 4,08E-05 | ERK |
| SNAI1 | Homo sapiens snail homolog 1 (Drosophila) (SNAI1), mRNA | 3,12 | 5,04E04 | ERK |
| MAP3K14 | Homo sapiens mitogen-activated protein kinase kinase kinase 14 (MAP3K14), mRNA | 1,99 | 4,88E-05 | NFκB |
| IL17RD | Homo sapiens interleukin 17 receptor D (IL17RD), mRNA | 1,95 | 3,13E-05 | |
| DAAM1 | Homo sapiens dishevelled associated activator of morphogenesis 1 (DAAM1), mRNA | 1,94 | 1,35E-05 | Wnt/JNK |
| IKBIP | Homo sapiens IKBKB interacting protein (IKBIP), transcript variant 2, mRNA | 1,86 | 3,96E-05 | NFκB |
| p38β | Homo sapiens mitogen-activated protein kinase 11 (MAPK11), mRNA | 1,80 | 8,95E-04 | p38 |
| IL10RA | Homo sapiens interleukin 10 receptor, alpha (IL10RA), transcript variant 1, mRNA | 1,79 | 7,08E-03 | |
| MAP3K3 | Homo sapiens mitogen-activated protein kinase kinase kinase 3 (MAP3K3), transcript variant 1, mRNA | 1,56 | 7,9E-05 | ERK/NFκB |
| IL8 | Homo sapiens interleukin 8 (IL8), mRNA | -1,56 | 5,98E-03 | |
| JUNB | Homo sapiens jun B proto-oncogene (JUNB), mRNA | -1,62 | 4,07E-02 | JNK |
| MAP3K4 | Homo sapiens mitogen-activated protein kinase kinase kinase 4 (MAP3K4), transcript variant 1, mRNA | -1,64 | 1,33E-03 | JNK |
| NFKB1 | Homo sapiens nuclear factor of kappa light polypeptide gene enhancer in B-cells 1 (NFKB1), transcript variant 1, mRNA | -2,07 | 1,35E-04 | NFκB |
| MMP7 | Homo sapiens matrix metallopeptidase 7 (matrilysin, uterine) (MMP7), mRNA | -2,11 | 9,96E-10 | |
| TLR4 | Homo sapiens toll-like receptor 4 (TLR4), transcript variant 1, mRNA | -2,21 | 3,54E-05 | JNK/NFκB |
| IFNGR2 | Homo sapiens interferon gamma receptor 2 (interferon gamma transducer 1) (IFNGR2), mRNA | -2,53 | 1,88E-04 | |
| FOSL1 | Homo sapiens FOS-like antigen 1 (FOSL1), mRNA | -2,54 | 2,36E-06 | JNK |
| CEBPD | Homo sapiens CCAAT/enhancer binding protein (C/EBP), delta (CEBPD), mRNA | -2,98 | 4,86E-04 | |
| LTB | Homo sapiens lymphotoxin beta (TNF superfamily, member 3) (LTB), transcript variant 1, mRNA | -4,78 | 9,85E-11 | |
Table S2

## Slide 3
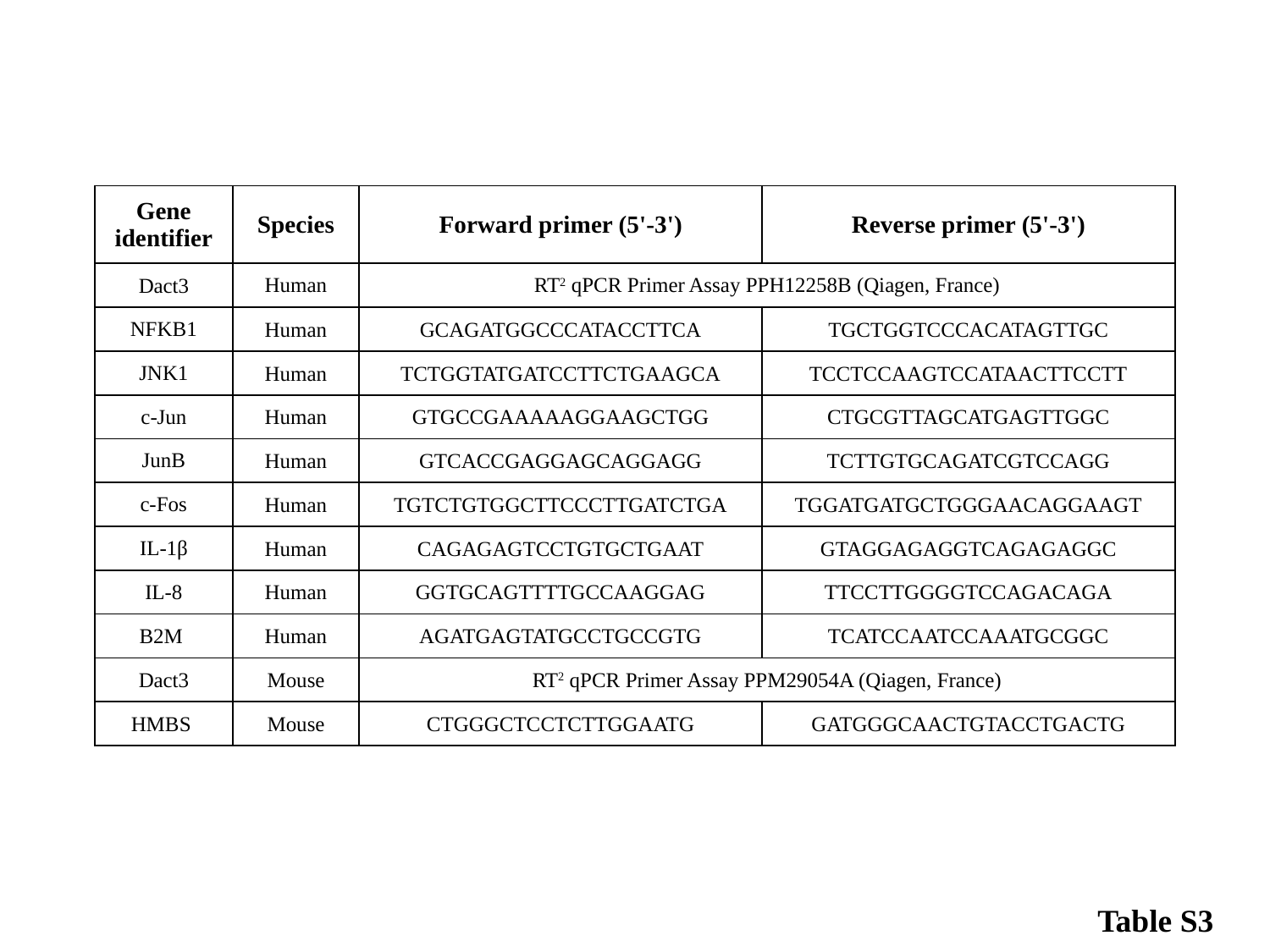

| Gene identifier | Species | Forward primer (5'-3') | Reverse primer (5'-3') |
| --- | --- | --- | --- |
| Dact3 | Human | RT2 qPCR Primer Assay PPH12258B (Qiagen, France) | |
| NFKB1 | Human | GCAGATGGCCCATACCTTCA | TGCTGGTCCCACATAGTTGC |
| JNK1 | Human | TCTGGTATGATCCTTCTGAAGCA | TCCTCCAAGTCCATAACTTCCTT |
| c-Jun | Human | GTGCCGAAAAAGGAAGCTGG | CTGCGTTAGCATGAGTTGGC |
| JunB | Human | GTCACCGAGGAGCAGGAGG | TCTTGTGCAGATCGTCCAGG |
| c-Fos | Human | TGTCTGTGGCTTCCCTTGATCTGA | TGGATGATGCTGGGAACAGGAAGT |
| IL-1β | Human | CAGAGAGTCCTGTGCTGAAT | GTAGGAGAGGTCAGAGAGGC |
| IL-8 | Human | GGTGCAGTTTTGCCAAGGAG | TTCCTTGGGGTCCAGACAGA |
| B2M | Human | AGATGAGTATGCCTGCCGTG | TCATCCAATCCAAATGCGGC |
| Dact3 | Mouse | RT2 qPCR Primer Assay PPM29054A (Qiagen, France) | |
| HMBS | Mouse | CTGGGCTCCTCTTGGAATG | GATGGGCAACTGTACCTGACTG |
Table S3

## Slide 4
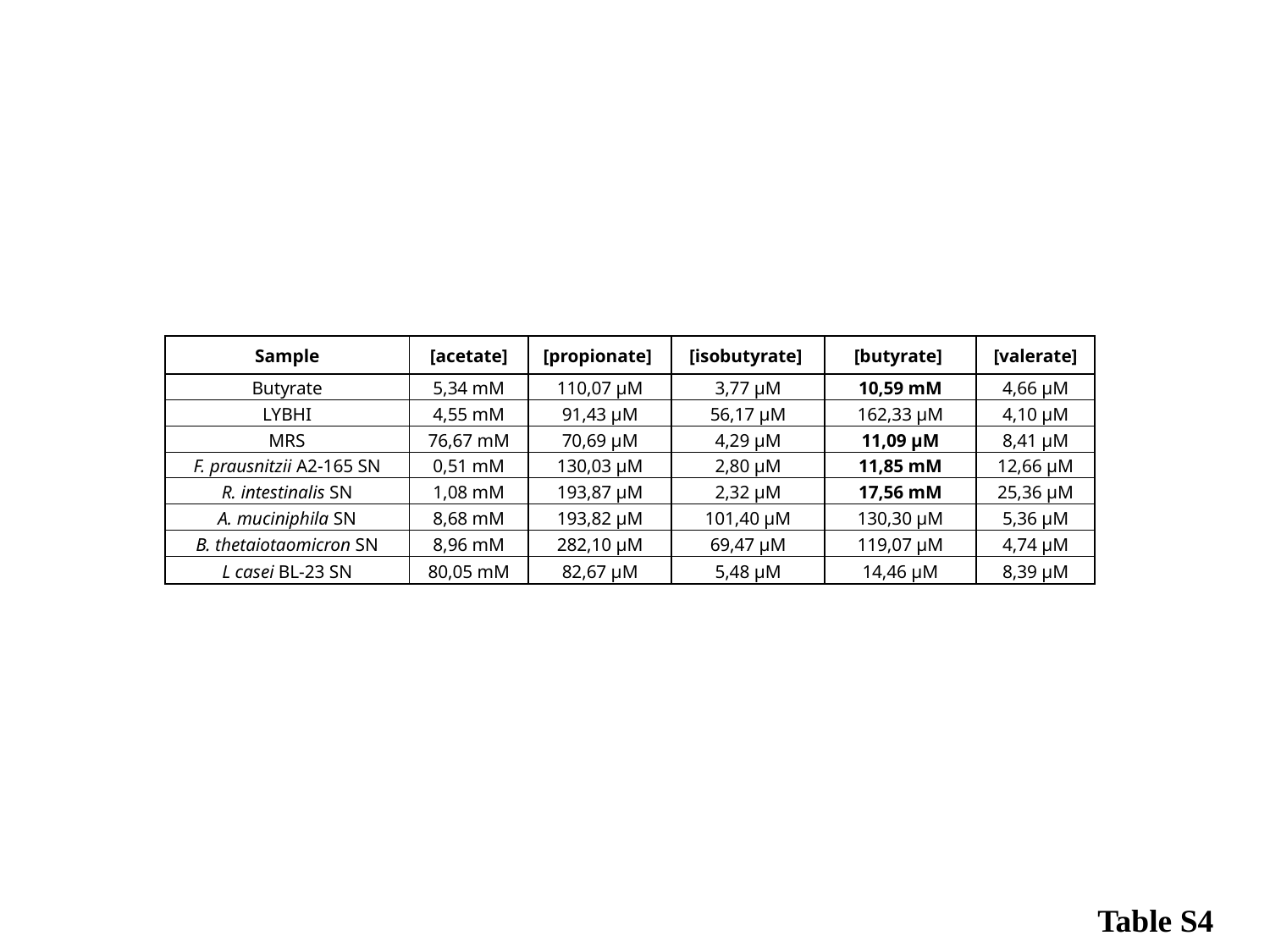

| Sample | [acetate] | [propionate] | [isobutyrate] | [butyrate] | [valerate] |
| --- | --- | --- | --- | --- | --- |
| Butyrate | 5,34 mM | 110,07 µM | 3,77 µM | 10,59 mM | 4,66 µM |
| LYBHI | 4,55 mM | 91,43 µM | 56,17 µM | 162,33 µM | 4,10 µM |
| MRS | 76,67 mM | 70,69 µM | 4,29 µM | 11,09 µM | 8,41 µM |
| F. prausnitzii A2-165 SN | 0,51 mM | 130,03 µM | 2,80 µM | 11,85 mM | 12,66 µM |
| R. intestinalis SN | 1,08 mM | 193,87 µM | 2,32 µM | 17,56 mM | 25,36 µM |
| A. muciniphila SN | 8,68 mM | 193,82 µM | 101,40 µM | 130,30 µM | 5,36 µM |
| B. thetaiotaomicron SN | 8,96 mM | 282,10 µM | 69,47 µM | 119,07 µM | 4,74 µM |
| L casei BL-23 SN | 80,05 mM | 82,67 µM | 5,48 µM | 14,46 µM | 8,39 µM |
Table S4
