# Supplementary figures and images for: Butyrate mediates anti-inflammatory effects of Faecalibacterium prausnitzii in intestinal epithelial cells through Dact3
Source: Gut Microbes. 2020 Oct 15;12(1):1826748. doi: 10.1080/19490976.2020.1826748 (PMC7567499; doi:10.1080/19490976.2020.1826748)

## Slide 1
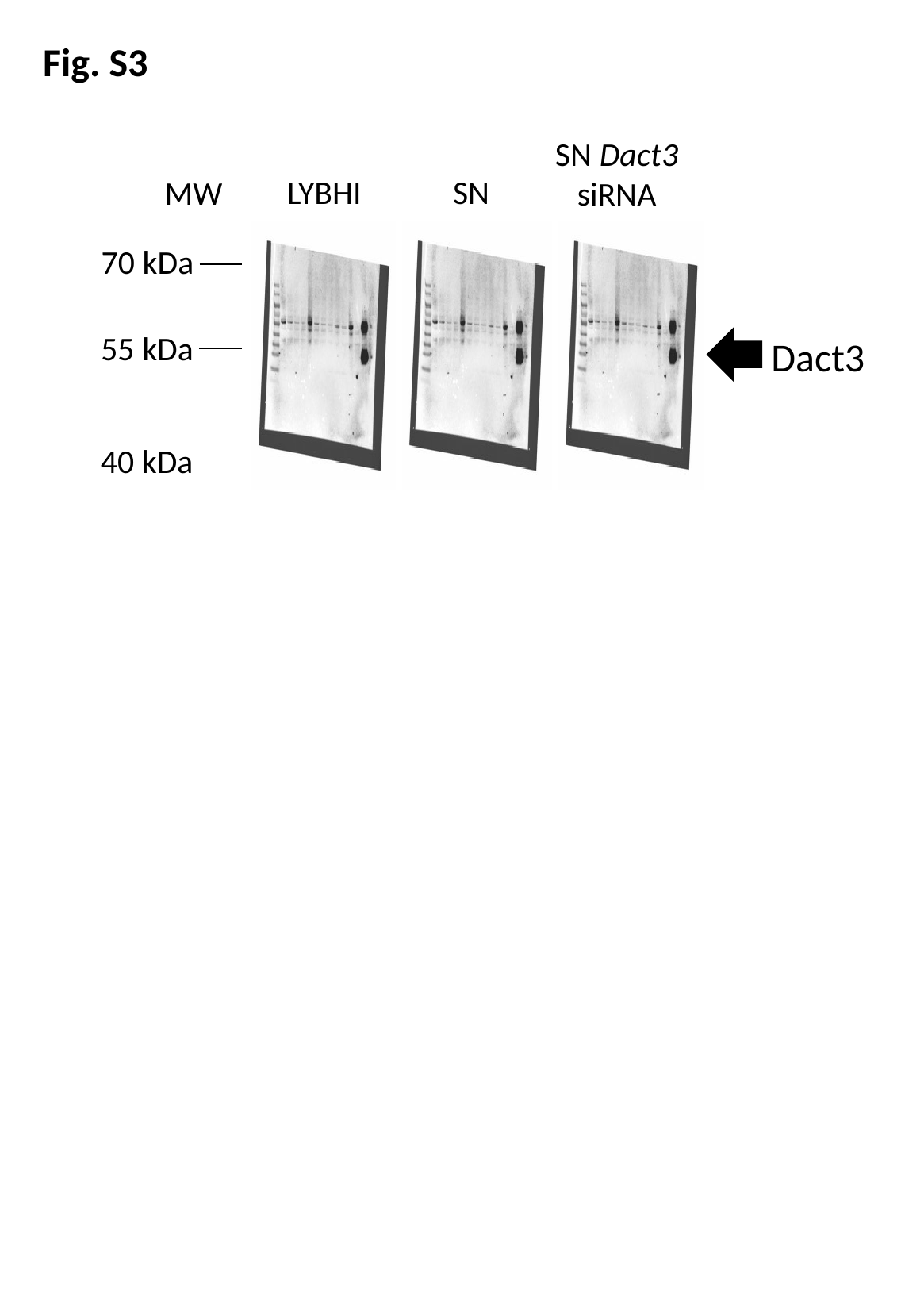

Fig. S3
SN Dact3 siRNA
SN
LYBHI
MW
70 kDa
55 kDa
Dact3
40 kDa

Supplement: Supplemental Material [file KGMI_A_1826748_SM0657.zip › Supplementary information/Fig S3 MS Dact3 Gut Microbes.pptx]

## Slide 1
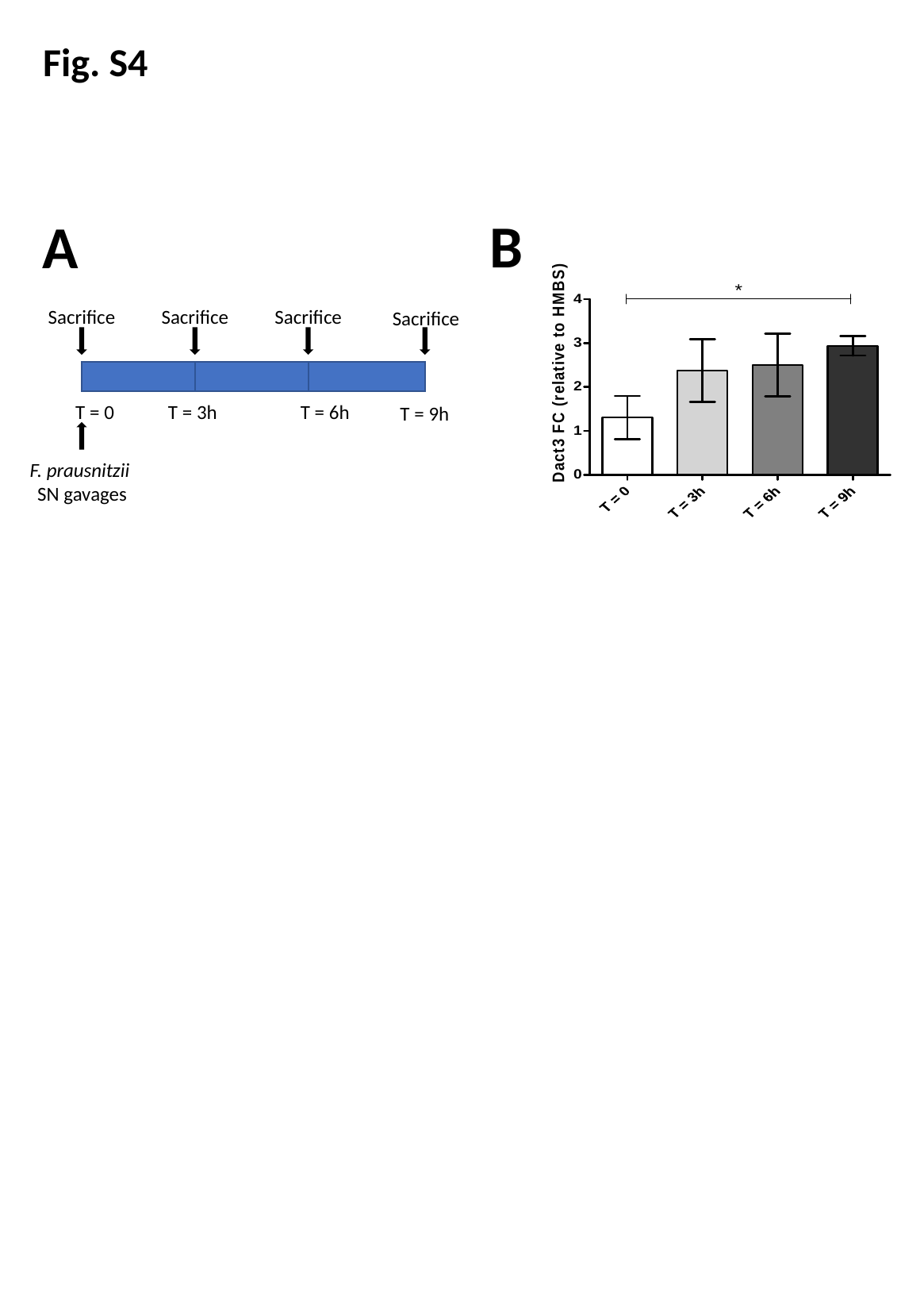

Fig. S4
B
A
Sacrifice
Sacrifice
Sacrifice
T = 0
T = 6h
T = 3h
F. prausnitzii
SN gavages
Sacrifice
T = 9h

Supplement: Supplemental Material [file KGMI_A_1826748_SM0657.zip › Supplementary information/Fig S4 MS Dact3 Gut Microbes.pptx]
